# A novel approach to intra-individual performance variability in ADHD

**DOI:** 10.1007/s00787-020-01555-y

**Published:** 2020-05-14

**Authors:** Annet Bluschke, Nicolas Zink, Moritz Mückschel, Veit Roessner, Christian Beste

**Affiliations:** Cognitive Neurophysiology, Department of Child and Adolescent Psychiatry, Faculty of Medicine, TU Dresden, Fetscherstrasse 74, 01307 Dresden, Germany

**Keywords:** Attention deficit hyperactivity disorder, Methylphenidate, Intra-individual variability

## Abstract

Patients with attention deficit/(hyperactivity) disorder (AD(H)D) show increased intra-individual variability (IIV) in behavioral performance. This likely reflects dopaminergic deficiencies. However, the precise performance profile across time and the pattern of fluctuations within it have not yet been considered, partly due to insufficient methods. Yet, such an analysis may yield important theory-based implications for clinical practice. Thus, in a case–control cross-sectional study, we introduce a new method to investigate performance fluctuations in patients with ADD (*n* = 76) and ADHD (*n* = 67) compared to healthy controls (*n* = 45) in a time estimation task. In addition, we also evaluate the effects of methylphenidate (MPH) treatment on this performance pattern in 29 patients with AD(H)D. Trial-by-trial differences in performance between healthy controls and patients with AD(H)D do not persist continuously over longer time periods. Periods during which no differences in performance between healthy controls and patients occur *alternate* with periods in which such differences are present. AD(H)D subtype and surprisingly also medication status does not affect this pattern. The presented findings likely reflect (phasic) deficiencies of the dopaminergic system in patients with AD(H)D which are not sufficiently ameliorated by first-line pharmacological treatment. The presented findings carry important clinical and scientific implications.

**Electronic supplementary material:**

The online version of this article (10.1007/s00787-020-01555-y) contains supplementary material, which is available to authorized users.

## Introduction

Attention deficit/(hyperactivity) disorder (AD(H)D) is a multi-faceted developmental disorder [1]. A hallmark of this disorder is an increased intra-individual variability (IIV) in behavioral performance, e.g., during reaction time (RT) tasks examining various cognitive functions [2–7]. In day-to-day life and in clinical practice, this becomes apparent as frequent and rapid attentional fluctuations which are characteristic of many children and adults with AD(H)D. From a scientific point of view, the phenomenon of IIV describes fluctuations in performance occurring after mastery (i.e., an expected level of performance) has been achieved. Increases within IIV, such as those frequently occurring within AD(H)D, indicate deficits in processing robustness, i.e., a reduced stability of performance across a period of time [8]. Since these aspects seem to be associated with symptom severity [9, 10], correlate with standardized measures of general cognitive skills [11] and may resemble an endophenotype of AD(H)D [2–4, 7, 12, 13], they are of considerable clinical importance.

**Fig. 1:**
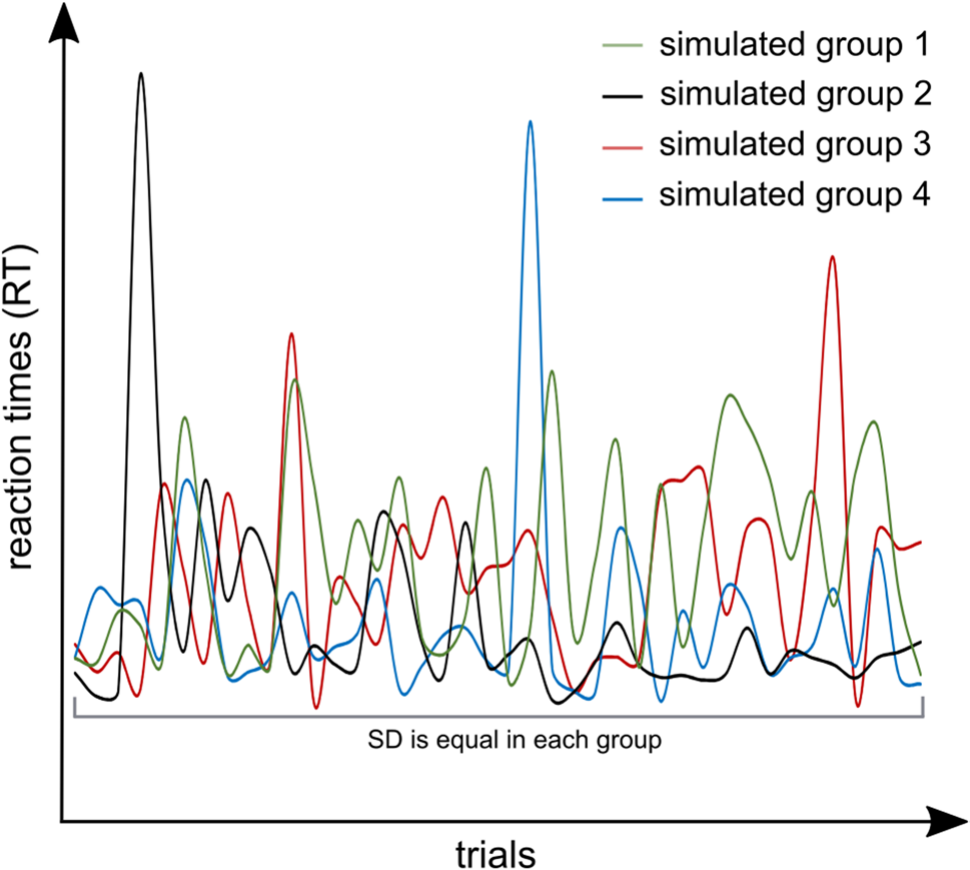
Simulation of different hypothetical performance profiles. All four simulated groups have the same standard deviation (SD) values. However, SD is not a sufficient indicator of performance fluctuations. Instead, the patterns underlying these differences in variability need to be considered and possible patterns within it need to be analyzed

Notably, even though it is the performance profile across time that constitutes IIV (i.e., how performance evolves on a trial-by-trial basis), this profile has not been considered in detail and cannot be captured using commonly applied measures of IIV (e.g., standard deviation of RTs) or by approaches examining different aspects of the *overall* distribution of RT data [3, 14]. In other words, established measures of IIV cannot depict whether IIV is constantly increased in AD(H)D or whether there are phases of increased IIV, followed by phases of non-increased IIV. In this case, only the phases of increased IIV would then constitute the generally increased IIV across time, which is captured by measures of overall RT distribution. Such a variable RT pattern would have important clinical implications for the treatment of patients with AD(H)D (see discussion section for examples). As exemplified in Fig. 1, fluctuations in performance could show various temporal profiles which implicate various patterns in the underlying information processing.

For example, it is possible that patients show high IIV because performance variations (i.e., fluctuations in RTs across time) are uniformly present throughout (refer Fig. 1, green line). Alternatively, it is possible that patients only show intermittent, yet very strong performance variations [15] (refer Fig. 1, black, red and blue lines). Crucially, these different patterns are not noticeable when using common measures to examine IIV (e.g., the SD of performance) as actually, all simulated examples shown in Fig. 1 have identical SDs (also see: [15]). However, the trial-by-trial performance profile carries important implications for clinical practice, as different patterns may require different behavioral adaptation strategies in daily life. Equally, the identification of any AD(H)D-specific differences in the performance pattern may provide highly relevant insights into the neurobiological foundations of this disorder. Here, it has been shown that deficiencies in the dopaminergic system underlie the emergence of a reduced stability of performance across a period of time/across trials [16]. Regarding neurobiology, especially striatal dopaminergic mechanisms are important for such processes [7, 17–19]. The likely reason is that reduced dopaminergic activity increases neuronal noise [20, 21], which leads to less distinct and unstable cortical representations resulting in decreases and variability in cognitive performance [16]. Theoretical [20, 22–24] and empirical research [24–29] underline the fact that dopaminergic modulation regulates the signal-to-noise ratio (SNR) of neural information processing. Based on the close relation between performance fluctuations and dopaminergic functions, the consideration of the trial-by-trial performance profile in patients with AD(H)D allows a closer insight into the nature of dopaminergic dysfunction in this patient group. If performance differences compared to healthy controls only occur intermittently, this would suggest that the dopaminergic system in AD(H)D fluctuates in its efficacy in reducing neuronal noise. However, this would also suggest that there is no general inability in AD(H)D to suppress neuronal noise and to perform on a stable level in cognitive tasks. Rather, this would suggest that there are transitory (phasic) dysfunctions in performance and therefore also likely in (phasic) dopaminergic neural transmission [15, 23].

Since time estimation processes are based upon mechanisms that are also central for the emergence of fluctuations in behavioral performance, they seem well suited for the examination of the trial-by-trial performance pattern in AD(H)D in comparison to healthy controls [30]. Overall, various aspects of timing including time estimation [31, 32], time discrimination [33], interval (re)production [3, 33], duration judgment and temporal set shifting [11] have previously been linked to ADHD (for overview, please see: [30]). It is especially interesting that time estimation processes have been shown to dissociate between patients with ADHD and patients with attention problems occurring due to the presence of other psychiatric disorders [11]. Conceptually, the time processing difficulties occurring in individuals with ADHD have been explained by pacemaker-counter models [34, 35], which suggest that time perception is driven by an opening/closing “switch” which allows for pulses generated by an “oscillating clock” to be counted [36]. A faster running “clock” would make time intervals seem longer and it has been suggested that this is the case in ADHD [37]. Timing problems in general have been closely linked to impulsivity, with neuroanatomical substrates substantially overlapping [17, 38–40]. Importantly, specifically task performance concerning shorter time spans (< 5 s) has been shown to be independent of attentional capacity [3]. Therefore, it is particularly useful to examine time estimation abilities in the second to millisecond time range in ADHD if aiming to obtain information not directly influenced by the level of attentional difficulties.

Since there have been some previous suggestions that time estimation processes [41] differ between the combined (ADHD) and inattentive (ADD) subtypes of AD(H)D [30], we subdivided the patient sample into two groups based on AD(H)D subtype. Here, we hypothesize finding significant group differences in the distribution of IIV across time when comparing either of the two patient groups to healthy controls. It is, however, unclear whether patients with ADD and ADHD will differ from each other.

Interestingly, recommended first-line pharmacological interventions using methylphenidate (MPH) [42–44] predominantly enhance tonic dopaminergic activity [45, 46]. Further, MPH may also somewhat suppress phasic aspects of dopaminergic neurotransmission [23, 29, 46–49]. MPH acts as a dopamine transporter (DAT) blocker [50, 51] which is highly expressed in the nigro-striatal and meso-corticolimbic pathways [52]. Because time estimation processes depend on striatal dopaminergic mechanisms [17–19, 30], MPH may be expected to modulate these processes [53]. Yet, as outlined above, it is possible that differences in performance between AD(H)D and healthy controls only occur intermittently. As MPH predominantly affects tonic dopaminergic activity [45–47, 54], it is possible that the trial-by-trial performance profile is only modulated to a limited extent by MPH treatment.

Importantly so far, the examination of the trial-by-trial performance has been hampered by insufficient methods to reliably examine how performance evolves on a trial-by-trial basis. To address this, we use a new methodological approach and apply a “trial-frequency of trials (TFT) decomposition” on the behavioral time series data; i.e., the series of all successive RTs. This approach is very similar to the approach taken by the widely used time–frequency analysis of neurophysiological data [55] and makes it possible to identify oscillatory patterns in behavior as it is usually done for EEG data (see “Methods” for details). Using this approach, it is possible to distinguish phases where IIV is increased as compared to healthy controls, and phases where IIV does not differ [15]. In the current study, we examined the performance of children with AD(H)D and healthy controls in a time estimation task, which has previously been shown to distinguish well between children with AD(H)D and healthy controls [41, 56]. Specifically, we compare healthy control children (i) to patients diagnosed with the inattentive subtype (ADD group) and (ii) to those diagnosed with the combined subtype (ADHD group). Here, we hypothesize that both patient groups will present with significantly lower accuracy rates than healthy controls. Further, groups are expected to present with different patterns of performance stability/reaction time variability in the TFT decomposition. Further, (iii) we conduct a comparison between the ADD group and the ADHD group. Here, some previous findings suggest that subgroup differences in time estimation and IIV may indeed occur, whereas others suggest no such differences (see above). Lastly, we examine (iv) the effects of MPH treatment on the performance profile across time and expect any differences between the control and patients groups to be reduced by treatment.

## Materials and methods

### Study design

**Fig. 2:**
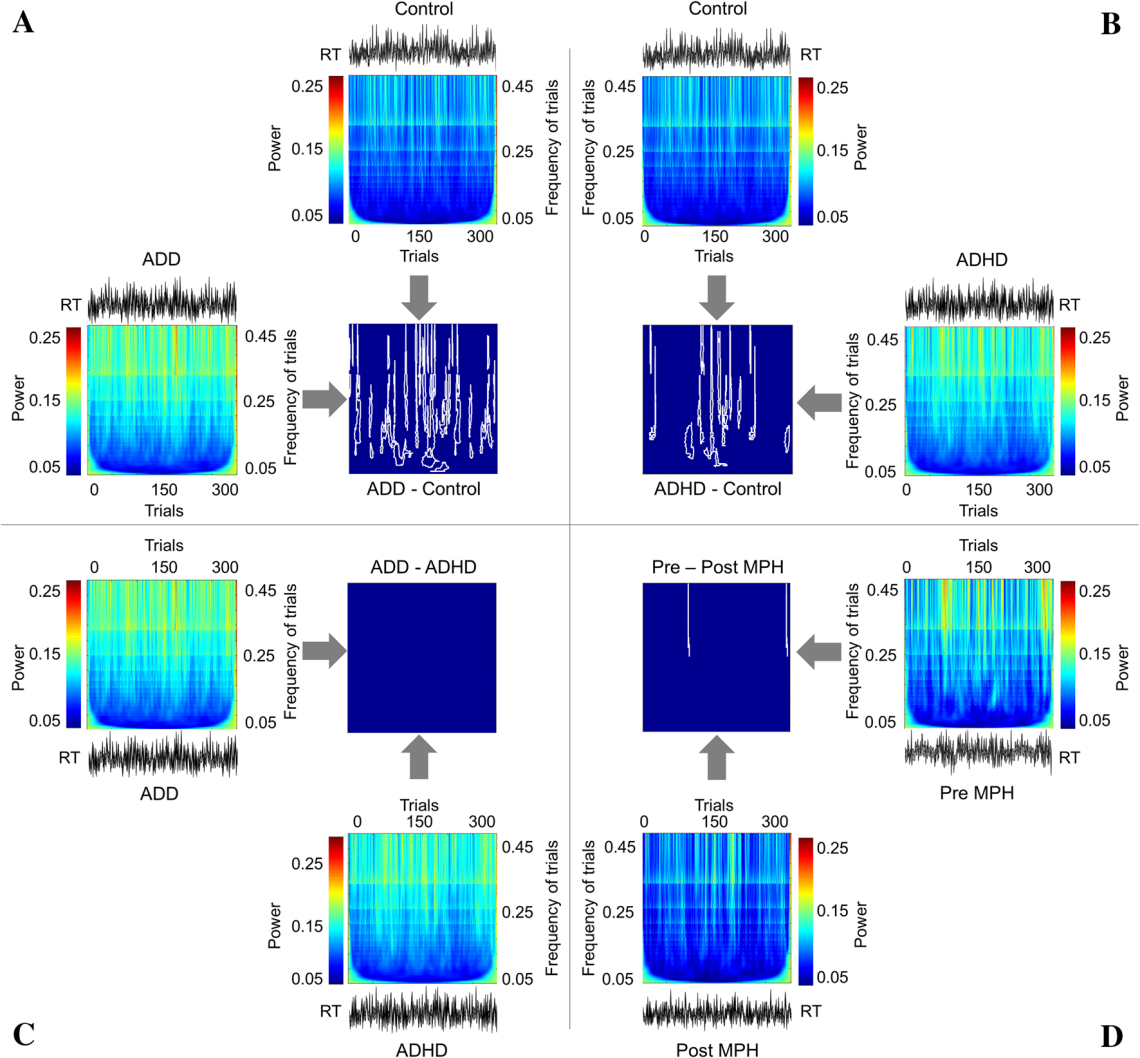
Results from the trial-by-trial analysis of reaction times plotted in 2D color-coded format where the power of RT variation is color coded, and vertical and horizontal axes represent frequency of trial and trials, respectively. RTs (black) are plotted on top or bottom of these 2D color plots. Results of comparison using cluster-based permutation tests are plotted in the center. White boundaries in these plots denote the clusters of trials in which significant group differences were evident. **a** The trial frequency of trials decomposed data is shown for the ADD (left) and the control group (top). The comparison of these groups is shown in the middle. **b** The ADHD (right) and the control group (top). The comparison of ADHD and control group is shown in the middle. **c** The ADD (left) and ADHD group (bottom). The comparison of ADD and ADHD is shown in the middle. **d** Data from the AD(H)D group prior (right) and post-MPH treatment (bottom). The comparison of pre-MPH and post-MPH treatment is shown in the middle

In a cross-sectional case–control study, we examined the trial-by-trial performance profiles of reaction times in a large sample of patients with AD(H)D compared to healthy controls. To achieve this, we used a novel analysis of RT data collected in a time estimation task. We compared healthy control children to patients diagnosed with the inattentive subtype (ADD group; Fig. 2a) and to those diagnosed with the combined subtype (ADHD group; Fig. 2b). Further, we conducted a comparison between the ADHD group and the ADD group (Fig. 2c). Lastly, we examined the effects of MPH treatment on the performance profile across time (Fig. 2d). Please not that an a priori sample size estimation was not possible since we did not focus on the use of traditional inferential statistics (incl. effect sizes).

### Participants

Only patients in whom a clinical AD(H)D diagnosis had been determined according to standard clinical guidelines by a team of experienced child and adolescent psychiatrists and psychologists in an outpatient clinic setting in the years 2015–2017 were considered in the study. The diagnostic procedure included family and school interviews, questionnaires, IQ (WISC-IV) and attention testing and the exclusion of possible somatic differential diagnoses via blood analyses, EEG, audiometry and vision testing. Following this extensive diagnostic procedure in the clinical setting, patient families were asked about their interest in taking part in this research study. Due to the recruitment context (outpatient clinic setting), it was not possible to record the number of participants who were not interested or able to take part in the study for various reasons. In this way, 143 patients in total could be recruited to take part in this study. *N* = 76 of them fulfilled clinical criteria for ADD according to ICD-10 (F98.8, subsequently referred to as the ADD sample), while the remaining *n* = 67 had been clinically diagnosed with the combined subtype (ADHD; ICD-10 F90.0 or F90.1, subsequently referred to as the ADHD sample). The patients were not affected by any further psychiatric comorbidity (e.g., ICD-10 codes F0-F7, F84 and F92-F95). Patients with additional conduct disorder or oppositional defiant disorder were only included if these symptoms were diagnostically seen as secondary to the ADHD core symptoms. 23 of the patients with ADD and 29 of those with ADHD were medicated with methylphenidate (range: 10–50 mg), but underwent a minimum of 24-h medication washout before study procedures. The minimum 24-h washout period was determined based on parent report and is the standard washout period used for stimulants given their short half-life [57, 58]. Medication status did not differ between the ADD and the ADHD group (*χ*^2^(1) = 2.6, *p* = 0.1). The healthy control sample consisted of *n* = 45 participants. Healthy controls were recruited from an internal participant database and by advertisements. The presence of AD(H)D was excluded through the use of child and parent questionnaires and an interview concerning ICD-10 diagnostic criteria. For an initial screening, telephone interviews were conducted 1–2 weeks before the appointment. During this interview, parents were interviewed about the diagnostic criteria of ADHD and were asked about the presence of any psychiatric symptoms of confirmed diagnoses of their child. In case of a good suitability for the study (i.e., agreement to participate by child and parent, fulfillment of all inclusion and of none of the exclusion criteria (see below)), questionnaires (ADHD symptom checklist) were sent to the families beforehand to be completed at home. Any items marked by parents as applicable to their child were discussed with the parents at the beginning of the appointment. A number of a priori inclusion and exclusion criteria were defined and applied to all participants. Participants were not included in the study if symptoms (or the presence of confirmed diagnoses) of severe or acute psychiatric disorders were reported during the telephone interview or within the symptom questionnaires (except for AD(H)D in the AD(H)D group). Further, they were excluded from the study if they reached an IQ score below 85 points (assessed by a short form of the WIC-IV, [59]), did not fall within the required age range of 8–15 years or had performed the time estimation task before. Please see Table 1 for demographic information.

**Table 1:** Demographics

	Healthy controls (*n* = 45)	Patients with add (*n* = 76)	Patients with ADHD (*n* = 67)	Pre-/post-mph (*n* = 29)	
Age	11.3 ± 2.2 years range: 8–15 years	10.8 ± 2.6 years range: 8–15 years	10.4 ± 1.9 years range: 8–15 years	10.2 ± 1.7 years range: 8–15 years	
IQ	IQ: 103 ± 12	IQ: 100 ± 11	IQ: 100 ± 14	IQ: 99.7 ± 12	
Gender	15f/30 m	10f/66 m	12f/55 m	3f/26 m	
Methylphenidate intake (24 h washout period prior to testing)	*n* = 0	*n* = 23	*n* = 29	Pre: *n* = 0	Post: *n* = 29
Inattention (stanine)	0.4 ± 0.2 (4.8 ± 1.9)	2.0 ± 0.44 (7.9 ± 1.0)	2.3 ± 0.47 (7.6 ± 1.7)	Pre: 2.0 ± 0.61	Post: 1.7 ± 0.59
Hyperactivity (stanine)	0.1 ± 0.2 (5.4 ± 0.6)	0.7 ± 0.44 (6.6 ± 1.0)	1.8 ± 0.59 (7.8 ± 1.3)	Pre: 1.2 ± 0.82	Post: 0.9 ± 0.75
Impulsivity (stanine)	0.3 ± 0.3 (5.3 ± 0.8)	1.0 ± 0.63 (6.8 ± 1.3)	2.3 ± 0.50 (7.9 ± 1.6)	Pre: 1.6 ± 0.9	Post: 1.4 ± 0.92

Table showing demographic data (m ± SD) (age, IQ as assessed by a short form of the WIC-IV [59], gender, symptom severity according to parent report as indicated on the AD(H)D Symptom Checklist (group averages and SDs of raw scores and stanine values) [60]) and details on medication treatment for all examined groups. For the single measurement in patients with ADD/ADHD, patients underwent a minimum of 24-h medication washout before study procedures. The minimum 24-h washout period was determined based on parent report and is the standard washout period used for stimulants given their short half-life [57, 58]. The last column shows the same data for the sample of patients tested twice (before (pre) and after (post) initiation of methylphenidate treatment)

This sample was compared to the ADD and the ADHD groups. Using the AD(H)D Symptom Checklist [60], parents rated their children on a scale of 0 (no problems) to 3 (severe problems) in regards to AD(H)D core symptoms (Table 1). Mean values above 1.5 indicate clinically severe symptoms [60]. Healthy controls had significantly lower scores than the two patient groups on all three subscales (all *F* > 139.8, all *p* < 0.001). Patients with ADHD and ADD did not differ significantly with regard to the degree of inattention (*p* = 0.12). As expected based on the different disorder characteristics, hyperactivity (*p* < 0.001) and impulsivity (*p* < 0.001) were significantly more pronounced in patients with ADHD than in those with ADD. Groups did not differ in age, IQ or gender distribution (all *F* < 1.6, all *p* > 0.4) (see Table 1).

Further, for *n* = 29 of the patients diagnosed with AD(H)D (see Table 1 for descriptives), performance on the time estimation task was examined a second time after treatment with MPH had been initiated (all drug-naïve beforehand). Initially, all patients received a low dose of immediate-release MPH and switched to extended-release MPH during the course of treatment. According to clinical guidelines, this dose was increased until (i) a significant and satisfactory symptom reduction was reported by parents or (ii) the target dose of 1 mg/kg body weight had been reached. Final doses ranged from 10 mg to 40 mg extended-release MPH per day (20.95 ± 8.6 mg/day). The initiation of MPH treatment in this group led to significant improvements in the domain of inattention (*t*(20) = 2.2; *p* = 0.04) and hyperactivity (*t*(20) = 2.1; *p* = 0.05), but not in regard to impulsivity (*t*(20) = 1.3; *p* = 0.2). Questionnaire data for either the pre- or the post-MPH time point was unfortunately missing. The study was approved by the local ethics committee. Informed written assent/consent was obtained from all participants/their legal guardians in accordance with the Declaration of Helsinki. The ethics committee of the Technical University of Dresden approved the study. Data can be made available upon reasonable request to the corresponding author.

### Task

Healthy controls and patients were asked to estimate a time of 1200 ms following a visual stimulus (white square on black background) [61]. They were asked to press a button whenever they thought that this time had elapsed. Responses given within 200 ms around this target time (i.e., between 1000 and 1400 ms) were accepted as correct. Responses given between 400 and 1000 ms after cue onset were classified as early responses. Responses occurring between 1400 and 2000 ms were classified as late answers. Any key press before 400 ms or after 2000 ms was classified as a missed response and was not included in further analyses. To have trade-off between ecological validity (e.g., such as demands in school environment) on the one hand, and isolating the processes of behavioral IIV on the other hand, visual feedback was given after every key press which requires using feedback learning to optimize performance. In the case of correct trials, a green happy smiley and the word “correct” were presented. In the case of early/late trials (including those later characterized as misses), a red sad smiley and the word “too early”/”too late” was presented. The words “did not react” were displayed if no response had occurred within 3000 ms after stimulus onset. Altogether, participants performed three blocks of 100 trials each. The intertrial interval was randomized between 800 and 2200 ms. Experimental performance was closely monitored online via an observation of the trigger codes occurring in the EEG recording software during the task.

### Statistical analysis of task performance

For the statistical analysis of task performance, response accuracy (i.e., the number of trials in which responses occurred within 200 ms around the target time of 1200 ms) and reaction times were compared between the groups. To analyze differences between healthy controls and the two AD(H)D subtype groups, we used a univariate analysis of variance (ANOVA) with the between-subject factor *Group* (healthy controls, ADD, ADHD). This was followed by Bonferroni-corrected post hoc tests where necessary. A paired samples t test was used to compare accuracy between testings with and without MPH.

### Statistical analysis of trial-by-trial performance profile

Gabor (Morlet) wavelets were used to analyze the performance (RT) profile across trials/time:

$$G\left( {t,f_{G} ,\sigma_{G} } \right) = \left( {1/\sqrt {2\pi } \sigma G} \right)\exp \left( {{{ - \Delta t^{2} } \mathord{\left/ {\vphantom {{ - \Delta t^{2} } {\left( {2\sigma_{G}^{2} } \right)}}} \right. \kern-0pt} {\left( {2\sigma_{G}^{2} } \right)}} + i2\pi f_{G} \Delta t} \right),$$

where *f*_*G*_ is its frequency, *σ*_*G*_ its amplitude standard deviation, and Δ*t* the “trial” deviation from the centre of the wavelet. The “size” of the wavelet is defined as its length in trial number and is usually represented in multiples of *σ*_*G*_; unless otherwise specified in the text, all wavelet values were *σ*_*G*_= 0.5/*f*_*G*_; the wavelet window was 4*σ*_*G*_; *f*_*G*_ is discretized in 1/*T *= 1/300 = 0.0033 steps where *T* is the length of all trials (here 300). The result of this procedure is plotted in 2D color-coded format where the power of RT variation is color-coded and vertical and horizontal axes represent the frequency of trial and trials, respectively (cf. Fig. 2). Because there is no a priori assumption concerning the trials at which the groups differ from each other (or MPH may affect performance), a data-driven strategy needed to be employed and corrected for multiple comparisons. This involves non-parametric cluster-based permutation testing [62] that is accomplished in two steps [63, 64] and requires two reference distributions: In the first step, t tests are calculated independently for each bin in the obtained map to test whether the measures in each group (patients, controls) could have been obtained from the same reference distribution (i.e., are not different from each other). For this, a distribution for each bin in the map is obtained by shuffling the data between the two conditions/groups *N*_shuffle_ = 5000 times. All significant time bins are then marked. The significance was set to *p* < 0.001. In the second step, clusters of contiguous significant bins are created and these data clusters are then compared to a reference distribution of clusters using a Monte Carlo randomization procedure of the original data (i.e., permutation testing). For this, a distribution for the cluster variable is computed that uses the result from the clusters of significant bins in each shuffled map and thus permits to create the reference distribution for clusters’ statistics. The significance level was set to *p* < 0.05. This procedure is used to compare the trial-by-trial performance profile across time between (i) the ADHD sample and the control sample, (ii) the ADD sample and the control sample, and (iii) the AD(H)D sample prior to MPH medication against this sample after 8 weeks of MPH medication had elapsed. These procedures were implemented in MATLAB using custom code [63] which can be downloaded at http://vision.ustc.edu.cn/packages/TutorialDataSetFunctions_TFanalysis.zip.

## Results

### Accuracy

Regarding the number of correct responses, the univariate ANOVA revealed a significant main effect of *Group* (*F*(2,185) = 21.78, *p *< 0.001, *η*_*p*_^*2*^= 0.19). Bonferroni-corrected post hoc tests showed that healthy executed significantly more responses in the target time interval than patients with ADD (*p *< 0.001) and those with ADHD (*p *< 0.001). Performance did not differ between the two AD(H)D subtypes (*p *> 0.99). Concerning the effect of MPH on correct responses, the applied paired t test revealed a significant improvement in the number of responses executed in the target time interval (*t*(28) = −5.2, *p *< 0.001).

Regarding the number of too early responses, a significant main effect of *Group* was revealed *F*(2,185) = 14.88, *p *< 0.001, *η*_*p*_^2^ = 0.14). Bonferroni-corrected post hoc tests showed that healthy controls executed significantly less responses prior to the target time interval than patients with ADD (*p* < 0.001) and those with ADHD (*p* < 0.001). Performance did not differ between the two AD(H)D subtypes (*p* > 0.99). Concerning the effect of MPH on the number of too early responses, paired t tests revealed a significant decrease in the number of too early responses (*t*(28) = 2.52, *p *= 0.018). As to the number of too late responses, the univariate ANOVA revealed a significant main effect of *Group* (*F*(2,185) = 3.80, *p *= 0.024, *η*_*p*_^*2*^= 0.39). Bonferroni-corrected post hoc tests showed that healthy controls executed significantly less responses after the target time interval than patients with ADD (*p *= 0.028). Yet, the number of too late responses did not differ between healthy controls and patients with ADHD (*p *= 0.075). Performance did not differ between the two AD(H)D subtypes (*p *> 0.99). Concerning the effect of MPH on the number of too late responses, paired t tests revealed a significant decrease in the number of too late responses (*t*(28) = 3.14, *p *= 0.004). The descriptive values can be found in Table 2.

**Table 2:** Behavioural data

	Healthy controls (*n* = 45)	Patients with ADD (*n* = 76)	Patients with ADHD (*n* = 67)	Pre-/post-MPH (*n* = 29)	
Correct responses	191.6 ± 35.9	133.6 ± 54.8	138.9 ± 50.7	134.7 ± 45.6	177.6 ± 50.4
Too early responses	57.6 ± 21	93.3 ± 43	93.9 ± 42.2	97.7 ± 44.7	74.8 ± 41.1
Too late responses	48.2 ± 24.2	61.6 ± 31.4	60.0 ± 23.1	61.2 ± 24.7	46.4 ± 21.3
Reaction time	1171 ± 50	1137 ± 115	1138 ± 89	1151 ± 61	1144 ± 88
Reaction time variability	247 ± 66	404 ± 171	382 ± 144	351 ± 127	280 ± 119

Accuracy (absolute numbers out of 300 trials of correct, too early and too late responses) and reaction time (variability) (in ms) (both given as mean ± SD) for the healthy controls (HC) and the ADD and ADHD groups and for the comparison between pre- and post-MPH (see “Participants” section for details)

### Reaction times

Regarding RTs, the univariate ANOVA revealed no significant main effect of *Group* (*F*(2,185) = 2.176, *p *= 0.116). However, the analysis of the standard deviation of the RTs (RT_SD_) showed a significant main effect of *Group* (*F*(2,185) = 18.55, *p *< 0.001, *η*_*p*_^2^ = 0.17). Further Bonferroni-corrected post hoc tests for RT_SD_ showed that patients with ADD (*p *< 0.001) and those with ADHD (*p* < 0.001) had significantly higher RT_SD_ as compared to healthy controls. Yet, no differences in RT_SD_ were found between ADD and ADHD patients (*t*(141) = −0.043, *p *= 0.966). Concerning the effect of MPH on accuracy, the applied paired t test revealed no difference in RTs (*t*(28) = 0.375, *p* = 0.713), but a significant difference in RT_SD_ (*t*(28) = −5.2, *p *< 0.001). Regarding the AD(H)D Symptom Checklist, RT_SD_ correlated with inattention (*r* = 0.309, *p *< 0.0001), moderately with hyperactivity (*r* = 0.192, *p* = 0.014), but not with impulsivity (*r *= 0.128, *p *= 0.105). The descriptive values can be found in Table 2.

However, the trial-to-trial performance across the entire task is of main interest for the current study and shown in Fig. 2.

In the center of Fig. 2, the results of the statistical comparison using cluster-based permutation tests are shown. Figure 2a shows the comparison between ADD patients and healthy controls. The comparison of the trial-by-trial performance (RT) profile across time between the ADD group and the control group revealed eleven clusters (significance level set to *p* < 0.05, see step 2 in “Methods”) in which trials showed significant differences in RTs between the ADD group and the control group (significance level set to *p* < 0.001, see step 2 in “Methods”). The comparison of ADHD patients and healthy controls is shown in Fig. 2b. The cluster-based permutation analysis revealed seven clusters (significance level set to *p* < 0.05, see step 2 in “Methods” section) in which RTs differed between ADHD group and healthy controls (significance level set to *p* < 0.001, see step 2 in “Methods” section). As can be seen in Fig. 2a, b, differences between healthy controls and AD(H)D subgroups do not occur continuously throughout the entire task. Rather, periods during which no differences in performance between healthy controls and patients were evident *alternate* with periods in which significant differences between groups were evident. For the ADD subgroup, 11 clusters containing a total of 68.7% of all trials showed differences as compared to the control group. For the ADHD subgroup, seven clusters comprising 40.7% of all trials showed differences as compared to the control group. The results of the cluster-based permutation analysis comparing ADD and ADHD subjects are shown in Fig. 2c. As can be seen, the performance profile comparison between ADD and ADHD group revealed that no clusters and no trials significantly differed between these groups. This means that there are no substantial differences in the pattern of trial-by-trial performance between ADD and ADHD patient groups. Figure 2d shows the comparison of ADD/ADHD patients before (pre-MPH) and after MPH medication (post-MPH). There were only two very small clusters in which trials differed (significance level set to *p* < 0.05, see step 2 in “Methods” section). Since this cluster only comprised six trials (2% of all trials), this suggests that no substantial differences are induced by MPH administration.

In sum, patients made less correct responses, which was due to a larger number of too early (ADD and ADHD) and too late (ADD) responses when compared with healthy controls. This response pattern is also reflected by a larger response time variability in both ADHD groups, although their mean response times were equal to the control group.

## Discussion

In the current study, we examined trial-by-trial performance profiles of reaction times in a large sample of patients with AD(H)D compared to healthy controls. To achieve this, we used a novel analysis of RT data collected in a time estimation task. We compared healthy control children to patients diagnosed with the inattentive subtype (ADD group; Fig. 2a) and to those diagnosed with the combined subtype (ADHD group; Fig. 2b). Further, we conducted a comparison between the ADD group and the ADHD group (Fig. 2c). Lastly, we examined the effects of MPH treatment on the performance profile across time in a subgroup of AD(H)D patients (Fig. 2d).

**Fig. 3:**
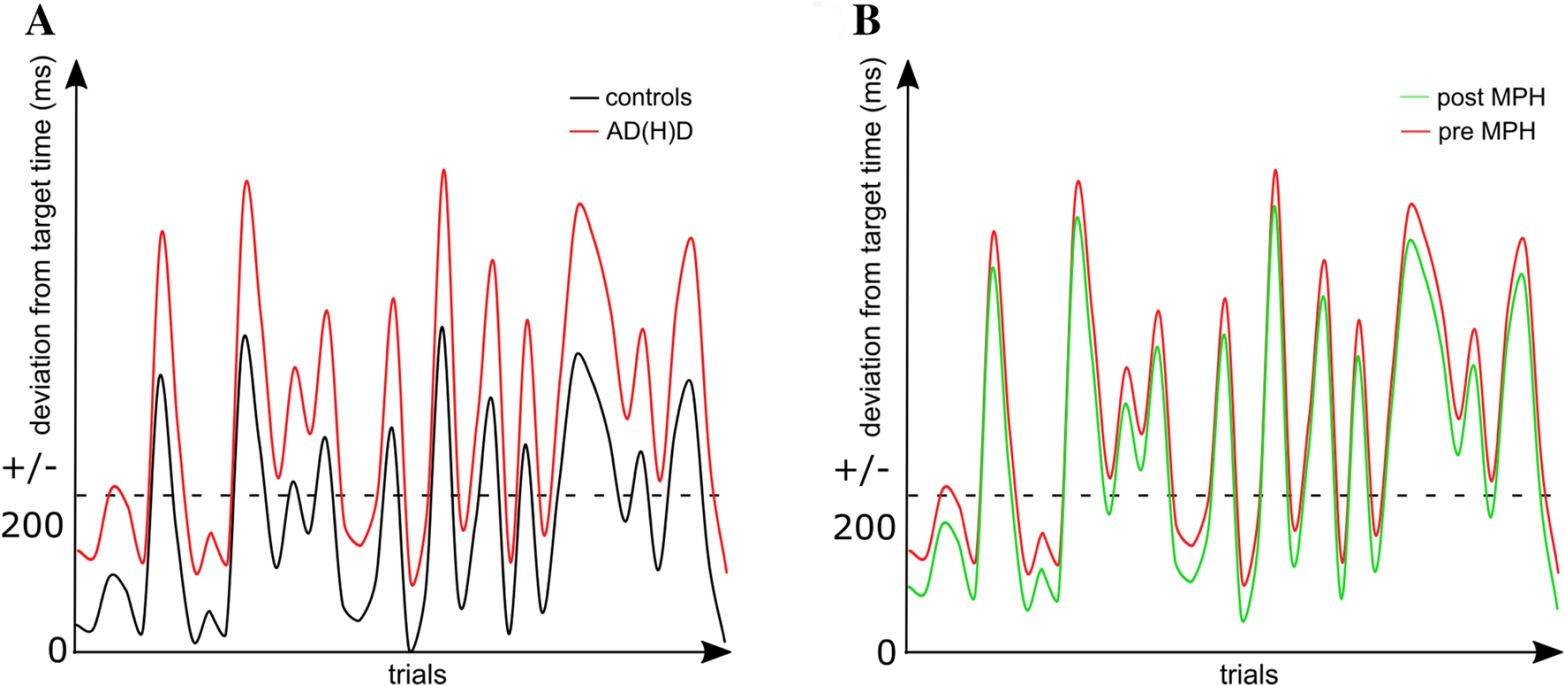
Simplified representation of the study results. **a** Healthy controls (black line) and AD(H)D patients (red line). **b** AD(H)D patients before (red line) and after treatment with methylphenidate (MPH) had been initiated (green line). Compared to performance without MPH treatment, only the overall level along the *y*-axis (downward shift) is changed compared to performance with MPH treatment. The amplitude of the fluctuations remains the same after MPH treatment

An analysis of the accuracy to respond within the target time interval revealed significant differences between healthy controls and patients with both AD(H)D subtypes. No differences were found between the inattentive (ADD) and combined subtype (ADHD) of AD(H)D. This pattern has previously been shown in examinations of reaction time variability [65]. Yet, the trial-to-trial performance profile was of main interest here. To aid the discussion, we kindly refer the reader to Fig. 3 which shows a simplified representation of the study results. Note that this figure does not contain the actually measured/decomposed data. The finding that patients with AD(H)D showed lower accuracy rates compared to healthy controls is shown in a general upward shift along the *y*-axis in Fig. 3a.

The analysis of the trial-by-trial performance profile shows that both patient groups differ significantly from the healthy controls (Fig. 2a, b). However, patients with ADD and ADHD did not differ from each other concerning the performance profile across trials in the time estimation task, which is in line with the overall accuracy data (Fig. 2c). This may be due to the fact that IIV and behavioral performance fluctuations are closely linked to inattention [65, 66], which patients of both subtypes have in common. Patients with AD(H)D generally showed lower accuracy rates compared to healthy controls. This is in line with previous findings [17, 31–33, 37, 53, 67].

**Fig. 4:**
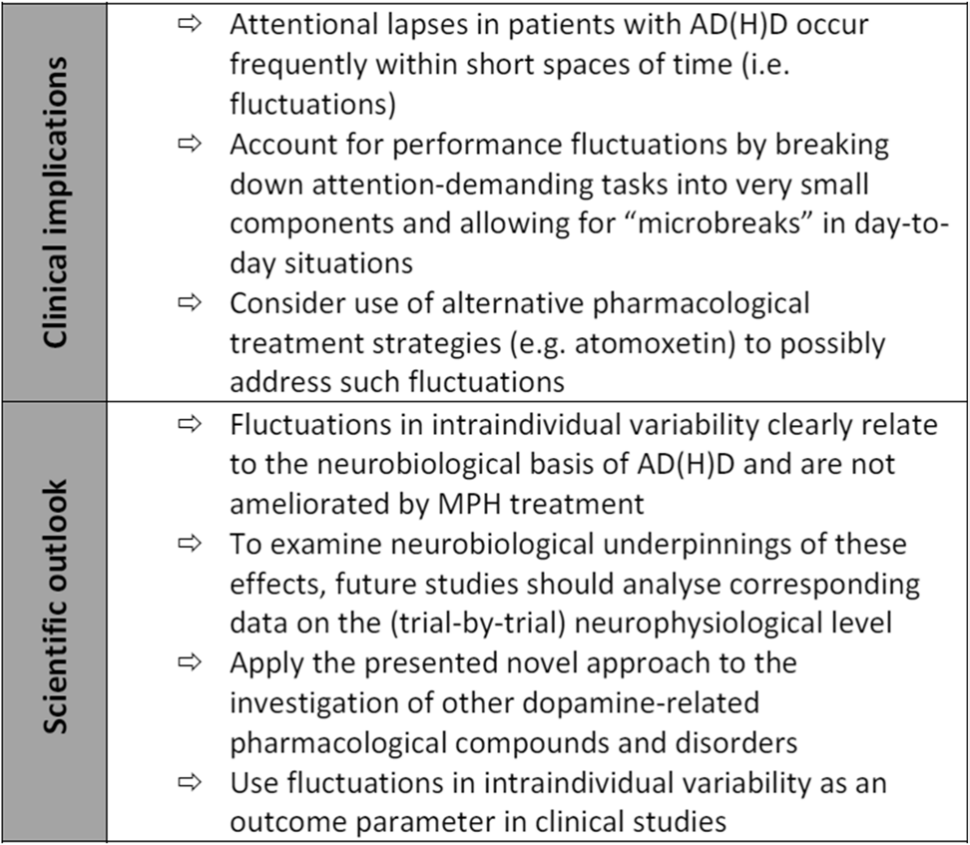
Clinical and scientific implications derived from the application of the presented novel methodological approach to analyze the behavioral performance profile in patients with AD(H)D

Overall, the results show that differences between patients with AD(H)D and healthy controls did not occur continuously throughout the entire task. Instead, the data show that performance was only different in some of the trials. Importantly, the results show a more fluctuating pattern of performance than it is the case in healthy controls (for an example illustration refer to Fig. 3a). That is, there are some periods during which no differences in performance between healthy controls and patients were evident. Importantly, these periods *alternate* with periods in which significant reaction time differences occur compared to healthy controls. These findings carry important clinical and scientific implications (see Fig. 4 for overview). From a clinical point of view, the results show that attention in children with AD(H)D may fluctuate rapidly across short spaces of time. Consequently, it may be useful to allow for “microbreaks” (i.e., off-task periods in the range of a few seconds) rather than aiming to achieve full on-task attention for prolonged periods of time. Such an approach has previously been studied in healthy adults, in whom microbreaks (e.g., lasting for 5 s [68] or 40 s [69]) have been shown to significantly improve sustained attention [68, 69], with this even being reflected in its neurophysiological correlates [68]. Further, breaking down longer tasks into microtasks has been shown to lead to increased task accuracy and more resilience to interruptions, albeit at the price of somewhat longer overall completion time [70]. Based on the current findings, such an approach may also prove useful in the case of AD(H)D, as it may match the fluctuating nature of attention very well. In fact, it may be possible that patients with AD(H)D use microbreaks (i.e., brief attentional lapses) as a self-regulatory strategy in order to reach required task demands [11]. To enhance its efficacy, such an approach may be combined with a token system to support the development of appropriate behavioral strategies. Importantly, this may be useful independent of AD(H)D subtype and also in patients already receiving treatment with MPH. Since recent evidence has shown divergent findings on periodicity of attentional fluctuations in patients with AD(H)D which do no not support the claim that that IIV in AD(H)D is the product of spontaneous periodic lapses of attention [71], more well-designed studies are needed to thoroughly examine the usefulness of such an approach in children with AD(H)D.

Furthermore, the current findings are crucial when considering the neurobiological basis of AD(H)D. Increased intra-subject variability of reaction times in AD(H)D has been hypothesized to reflect interference from the default mode network (DMN) [72–77] and dopaminergic modulation [78–83]. Deficiencies in the dopaminergic system are important for the emergence of a reduced stability of performance across a period of time [16]. The possible reason is that reduced dopamine activity increases neuronal noise [16, 20], which leads to less distinct and less stable cortical representations resulting in decreases in cognitive performance and increases in IIV [16]. Several lines of evidence suggest that dopaminergic modulation is a mechanism regulating the signal-to-noise ratio (SNR) of neural information processing [20, 22, 24, 29]. Considering this evidence, the current results suggest that the dopaminergic system in AD(H)D is just partly able to suppress neuronal noise. This seems to occur independent of AD(H)D subtype. It seems that periods during which neuronal noise is successfully suppressed alternate with periods during which this does not occur. Consequently, performance becomes different to that of healthy controls. It therefore seems that there are fluctuations in the efficacy of the dopaminergic system to suppress neuronal noise in patients with AD(H)D.

Regarding the above interpretation that the observed differences between AD(H)D and healthy controls may reflect dysfunctions in dopamine neurotransmission, the observed findings on MPH effects are important. Overall, patients performed more accurately after 8 weeks of MPH administration. Yet, there were no systematic and significant differences in the trial-by-trial performance profile across the duration of the task. Indeed, a previous study also failed to show significant effects of MPH on a variability measure in a sustained attention task [84]. Crucially, MPH mainly affects tonic dopaminergic activity [45, 46]. The obtained data show that only overall accuracy, but not trial-by-trial fluctuations, is affected by that. This result is reflected in the general downward shift along the y-axis together with the constantly high amplitudes after initiating MPH treatment in the hypothetical model shown in Fig. 3b. The finding of increased overall performance accuracy, yet unchanged trial-by-trial fluctuations patterns may seem paradoxical but can be explained as follows (refer Fig. 3b).

MPH acts as a dopamine transporter (DAT) blocker and steadily modulates the reuptake of dopamine [50, 51]. This is why MPH predominantly affects tonic dopaminergic activity [45]. The task used in the current study required participants to estimate a time interval of 1200 ms. Responses were considered as in time when they were executed within 200 ms of this target time (Fig. 3). If the RT curve across trials is only slightly and monotonically shifted downward the y-axis (green line in Fig. 3b), significantly more responses occur within the required time range, leading to a larger number of correct (in-time) responses. Importantly, and as can be seen in Fig. 3b, this is possible without affecting the degree of fluctuation or the RT profile across trials (i.e., the amplitude modulation across trials). It is therefore possible that MPH may affect overall task performance (i.e., the number of in-time reactions) without affecting the degree of trial-by-trial fluctuations. The interpretation of the monotonic shift of the RT profile is in line with neurobiological processes associated with MPH effects [85]. The pattern of results suggests that trial-by-trial fluctuations in performance are largely independent of tonic modulations of dopaminergic activity as induced using the first-line pharmacological treatment in ADHD (i.e., MPH). This interpretation is in line with several findings suggesting that MPH predominantly affects tonic dopaminergic activity [45, 46]. Findings concerning the effects of MPH on phasic dopamine release are mixed [46, 47, 54] and the effects of MPH on phasic release seem to depend on the administered dose [23]. Especially therapeutic doses of MPH as used in the treatment of AD(H)D have been suggested to decrease the ratio between phasic and tonic dopamine [23]. Since MPH medication emphasizes tonic dopaminergic turnover, general performance levels are increased [23]. However, as the current study shows, this is not sufficient, as fluctuations in performance, which also represent a particular challenge in everyday functioning of AD(H)D patients [9, 10], are not improved by MPH. Such improvements in phasic dopaminergic neurotransmission may be achieved through other approaches to AD(H)D treatment. Phasic effects of dopaminergic activity are known to be mediated via (prefrontal) dopamine D1 receptors [86, 87]. Interestingly, atomoxetine, which is currently used as a second-line pharmacological approach to AD(H)D, has been shown to decrease “noise” in neuronal signaling by indirectly increasing dopamine stimulation of D1 and norepinephrine receptors [88, 89]. Since it is the modulation of neuronal noise that is central for the stability/variability of performance [16], it is possible that atomoxetine, which is equally potent as MPH to treat ADHD [90, 91], also affects trial-by-trial variability in ADHD. However, this clinical implication remains to be tested in future studies, since MPH administration was not counterbalanced and all subjects were on medication at the second time of measurement, MPH effects cannot be disentangled from effects of practice. Similarly, it will be important to examine trial-by-trial fluctuations in other cognitive domains and in other disorders associated with deficits in dopaminergic neurotransmission. Also, the analysis of possible corresponding patterns on the (trial-by-trial) neurophysiological level would be an important next step. Moreover, the current findings may be limited, since the task was adapted from Beste et al. [61], who conducted a study in patients with a neurological disorder (Huntington’s Disease) in adults rather than a child and adolescent sample with cognitive alterations related to AD(H)D. Future studies should also address the differential effects of response feedback on the attentional deficit subtypes (ADD vs ADHD).

Further, the generalizability of the results from the experimental to the clinical context remains to be elucidated. In this regard, it will be important for future studies to further tease apart the contributions of time estimation and feedback learning to timing skills in patients with ADHD.

In summary, the study examined IIV with a novel focus on the precise trial-by-trial performance profile. This has not been considered in detail until now. A new method/tool to examine the trial-by-trial behavioral performance profile in patients with AD(H)D in comparison to healthy controls and its relation to methylphenidate (MPH) treatment is presented. Findings show that there are periods during which no differences in performance between healthy controls and patients are present. These periods *alternate* with periods in which significant reaction time differences are found. Overall, however, the results point to phasic dopaminergic dysfunctions in AD(H)D that need to be more considered in treatment. They also carry possible important clinical implications which remain to be addressed in further studies.

## Electronic supplementary material

Below is the link to the electronic supplementary material.

Supplementary material 1 (DOCX 12 kb)

## References

[1] Kieling R, Rohde LA (2012). ADHD in children and adults: diagnosis and prognosis. Curr Top Behav Neurosci.

[2] Henríquez-Henríquez MP, Billeke P, Henríquez H (2014). Intra-individual response variability assessed by ex-Gaussian analysis may be a new endophenotype for attention-deficit/hyperactivity disorder. Front Psychiatry.

[3] Lin H-Y, Hwang-Gu S-L, Gau SS-F (2015). Intra-individual reaction time variability based on ex-Gaussian distribution as a potential endophenotype for attention-deficit/hyperactivity disorder. Acta Psychiatry Scand.

[4] Saville CWN, Feige B, Kluckert C (2015). Increased reaction time variability in attention-deficit hyperactivity disorder as a response-related phenomenon: evidence from single-trial event-related potentials. J Child Psychol Psychiatry.

[5] Rosch KS, Dirlikov B, Mostofsky SH (2013). Increased intrasubject variability in boys with ADHD across tests of motor and cognitive control. J Abnorm Child Psychol.

[6] Kofler MJ, Rapport MD, Sarver DE (2013). Reaction time variability in ADHD: a meta-analytic review of 319 studies. Clin Psychol Rev.

[7] Kuntsi J, Klein C (2012). Intraindividual variability in ADHD and its implications for research of causal links. Curr Top Behav Neurosci.

[8] Li S-C, Huxhold O, Schmiedek F (2004). Aging and attenuated processing robustness. GER.

[9] Sjöwall D, Thorell LB (2014). Functional impairments in attention deficit hyperactivity disorder: the mediating role of neuropsychological functioning. Dev Neuropsychol.

[10] van Lieshout M, Luman M, Twisk JWR (2017). Neurocognitive predictors of ADHD outcome: a 6-year follow-up study. J Abnorm Child Psychol.

[11] Walg M, Hapfelmeier G, El-Wahsch D, Prior H (2017). The faster internal clock in ADHD is related to lower processing speed: WISC-IV profile analyses and time estimation tasks facilitate the distinction between real ADHD and pseudo-ADHD. Eur Child Adolesc Psychiatry.

[12] Bluschke A, Chmielewski WX, Mückschel M (2017). Neuronal intra-individual variability masks response selection differences between ADHD subtypes-a need to change perspectives. Front Hum Neurosci.

[13] Bluschke A, Gohil K, Petzold M (2018). Neural mechanisms underlying successful and deficient multi-component behavior in early adolescent ADHD. Neuroimage Clin.

[14] Karalunas SL, Huang-Pollock CL, Nigg JT (2013). Is reaction time variability in ADHD mainly at low frequencies?. J Child Psychol Psychiatry.

[15] Gilden DL, Hancock H (2007). Response variability in attention-deficit disorders. Psychol Sci.

[16] MacDonald SWS, Nyberg L, Bäckman L (2006). Intra-individual variability in behavior: links to brain structure, neurotransmission and neuronal activity. Trends Neurosci.

[17] Coull JT, Cheng R-K, Meck WH (2011). Neuroanatomical and neurochemical substrates of timing. Neuropsychopharmacology.

[18] Merchant H, de Lafuente V (2014). Introduction to the neurobiology of interval timing. Adv Exp Med Biol.

[19] Petter EA, Lusk NA, Hesslow G, Meck WH (2016). Interactive roles of the cerebellum and striatum in sub-second and supra-second timing: support for an initiation, continuation, adjustment, and termination (ICAT) model of temporal processing. Neurosci Biobehav Rev.

[20] Li SC, Lindenberger U, Sikström S (2001). Aging cognition: from neuromodulation to representation. Trends Cogn Sci (Regul Ed).

[21] MacDonald SWS, Cervenka S, Farde L (2009). Extrastriatal dopamine D2 receptor binding modulates intraindividual variability in episodic recognition and executive functioning. Neuropsychologia.

[22] Servan-Schreiber D, Printz H, Cohen JD (1990). A network model of catecholamine effects: gain, signal-to-noise ratio, and behavior. Science.

[23] Sikström S, Söderlund G (2007). Stimulus-dependent dopamine release in attention-deficit/hyperactivity disorder. Psychol Rev.

[24] Ziegler S, Pedersen ML, Mowinckel AM, Biele G (2016). Modelling ADHD: a review of ADHD theories through their predictions for computational models of decision-making and reinforcement learning. Neurosci Biobehav Rev.

[25] Adelhöfer N, Gohil K, Passow S (2018). The system-neurophysiological basis for how methylphenidate modulates perceptual-attentional conflicts during auditory processing. Hum Brain Mapp.

[26] Beste C, Adelhöfer N, Gohil K (2018). Dopamine modulates the efficiency of sensory evidence accumulation during perceptual decision making. Int J Neuropsychopharmacol.

[27] Bluschke A, Friedrich J, Schreiter ML (2018). A comparative study on the neurophysiological mechanisms underlying effects of methylphenidate and neurofeedback on inhibitory control in attention deficit hyperactivity disorder. Neuroimage Clin.

[28] Pertermann M, Bluschke A, Roessner V, Beste C (2019). The modulation of neural noise underlies the effectiveness of methylphenidate treatment in attention-deficit/hyperactivity disorder. Biol Psychiatry Cogn Neurosci Neuroimaging.

[29] Yousif N, Fu RZ, Abou-El-Ela Bourquin B (2016). Dopamine activation preserves visual motion perception despite noise interference of human V5/MT. J Neurosci.

[30] Noreika V, Falter CM, Rubia K (2013). Timing deficits in attention-deficit/hyperactivity disorder (ADHD): evidence from neurocognitive and neuroimaging studies. Neuropsychologia.

[31] Pretus C, Picado M, Ramos-Quiroga A (2016). Presence of distractor improves time estimation performance in an adult ADHD sample. J Atten Disord.

[32] Wilson TW, Heinrichs-Graham E, White ML (2013). Estimating the passage of minutes: deviant oscillatory frontal activity in medicated and unmedicated ADHD. Neuropsychology.

[33] Smith A, Taylor E, Rogers JW (2002). Evidence for a pure time perception deficit in children with ADHD. J Child Psychol Psychiatry.

[34] Buhusi CV, Meck WH (2009). Relativity theory and time perception: single or multiple clocks?. PLoS ONE.

[35] Buhusi CV, Meck WH (2005). What makes us tick? Functional and neural mechanisms of interval timing. Nat Rev Neurosci.

[36] Lake JI, LaBar KS, Meck WH (2016). Emotional modulation of interval timing and time perception. Neurosci Biobehav Rev.

[37] Walg M, Oepen J, Prior H (2015). Adjustment of time perception in the range of seconds and milliseconds: the nature of time-processing alterations in children with ADHD. J Atten Disord.

[38] Rubia K, Halari R, Christakou A, Taylor E (2009). Impulsiveness as a timing disturbance: neurocognitive abnormalities in attention-deficit hyperactivity disorder during temporal processes and normalization with methylphenidate. Philos Trans R Soc Lond B Biol Sci.

[39] Coull JT, Vidal F, Nazarian B, Macar F (2004). Functional anatomy of the attentional modulation of time estimation. Science.

[40] Coull JT, Nobre AC (1998). Where and when to pay attention: the neural systems for directing attention to spatial locations and to time intervals as revealed by both PET and fMRI. J Neurosci.

[41] Bluschke A, Schuster J, Roessner V, Beste C (2018). Neurophysiological mechanisms of interval timing dissociate inattentive and combined ADHD subtypes. Sci Rep.

[42] Childress AC, Sallee FR (2014). Attention-deficit/hyperactivity disorder with inadequate response to stimulants: approaches to management. CNS Drugs.

[43] Mattingly GW, Wilson J, Rostain AL (2017). A clinician’s guide to ADHD treatment options. Postgrad Med.

[44] American Academy of Pediatrics (2011). ADHD: clinical practice guideline for the diagnosis, evaluation, and treatment of attention-deficit/hyperactivity disorder in children and adolescents. Pediatrics.

[45] Badgaiyan RD, Sinha S, Sajjad M, Wack DS (2015). Attenuated tonic and enhanced phasic release of dopamine in attention deficit hyperactivity disorder. PLoS ONE.

[46] Engert V, Pruessner JC (2008). Dopaminergic and noradrenergic contributions to functionality in ADHD: the role of methylphenidate. Curr Neuropharmacol.

[47] Evers EA, Stiers P, Ramaekers JG (2017). High reward expectancy during methylphenidate depresses the dopaminergic response to gain and loss. Soc Cogn Affect Neurosci.

[48] Volkow ND, Wang G-J, Fowler JS, Ding Y-S (2005). Imaging the effects of methylphenidate on brain dopamine: new model on its therapeutic actions for attention-deficit/hyperactivity disorder. Biol Psychiatry.

[49] Seeman P, Madras B (2002). Methylphenidate elevates resting dopamine which lowers the impulse-triggered release of dopamine: a hypothesis. Behav Brain Res.

[50] Skirrow C, McLoughlin G, Banaschewski T (2015). Normalisation of frontal theta activity following methylphenidate treatment in adult attention-deficit/hyperactivity disorder. Eur Neuropsychopharmacol.

[51] Volkow ND, Wang GJ, Fowler JS (1999). Methylphenidate and cocaine have a similar in vivo potency to block dopamine transporters in the human brain. Life Sci.

[52] Ciliax BJ, Drash GW, Staley JK (1999). Immunocytochemical localization of the dopamine transporter in human brain. J Comp Neurol.

[53] Smith A, Cubillo A, Barrett N (2013). Neurofunctional effects of methylphenidate and atomoxetine in boys with attention-deficit/hyperactivity disorder during time discrimination. Biol Psychiatry.

[54] Cools R, Barker RA, Sahakian BJ, Robbins TW (2001). Enhanced or impaired cognitive function in Parkinson’s disease as a function of dopaminergic medication and task demands. Cereb Cortex.

[55] Herrmann CS, Rach S, Vosskuhl J, Strüber D (2014). Time-frequency analysis of event-related potentials: a brief tutorial. Brain Topogr.

[56] Vahid A, Bluschke A, Roessner V (2019). Deep learning based on event-related eeg differentiates children with ADHD from healthy controls. J Clin Med.

[57] Isiten HN, Cebi M, Sutcubasi Kaya B (2017). Medication effects on EEG biomarkers in attention-deficit/hyperactivity disorder. Clin EEG Neurosci.

[58] Wigal SB, Gupta S, Greenhill L (2007). Pharmacokinetics of methylphenidate in preschoolers with attention-deficit/hyperactivity disorder. J Child Adolesc Psychopharmacol.

[59] Waldmann H-C (2008). Kurzformen des HAWIK-IV: Statistische Bewertung in verschiedenen Anwendungsszenarien. Diagnostica.

[60] Döpfner M, Görtz-Dorten A, Lehmkuhl G (2008). Diagnostik-System für Psychische Störungen im Kindes- und Jugendalter nach ICD-10 und DSM-IV, DISYPS-II.

[61] Beste C, Saft C, Andrich J (2007). Time processing in Huntington’s disease: a group-control study. PLoS ONE.

[62] Maris E, Oostenveld R (2007). Nonparametric statistical testing of EEG- and MEG-data. J Neurosci Methods.

[63] Beste C, Kaping D, Tzvetanov T (2018) Extension of the non-parametric cluster-based time-frequency statistics to the full time windows and to single condition tests. arXiv:180109372 [q-bio]

[64] Maris E (2012). Statistical testing in electrophysiological studies. Psychophysiology.

[65] Kuntsi J, Pinto R, Price TS (2014). The separation of ADHD inattention and hyperactivity-impulsivity symptoms: pathways from genetic effects to cognitive impairments and symptoms. J Abnorm Child Psychol.

[66] Adams ZW, Roberts WM, Milich R, Fillmore MT (2011). Does response variability predict distractibility among adults with attention-deficit/hyperactivity disorder?. Psychol Assess.

[67] Hwang S-L, Gau SS-F, Hsu W-Y, Wu Y-Y (2010). Deficits in interval timing measured by the dual-task paradigm among children and adolescents with attention-deficit/hyperactivity disorder. J Child Psychol Psychiatry.

[68] Mijović P, Ković V, Mačužić I (2015). Do micro-breaks increase the attention level of an assembly worker? An ERP study. Procedia Manuf.

[69] Lee KE, Williams KJH, Sargent LD (2015). 40-second green roof views sustain attention: the role of micro-breaks in attention restoration. J Environ Psychol.

[70] Cheng J, Teevan J, Iqbal ST, Bernstein MS (2015) Break it down: a comparison of macro- and microtasks. In: Proceedings of the 33rd annual ACM conference on human factors in computing systems—CHI’15, pp 4061–4064. ACM Press, Seoul

[71] Salum GA, Sato JR, Manfro AG (2019). Reaction time variability and attention-deficit/hyperactivity disorder: is increased reaction time variability specific to attention-deficit/hyperactivity disorder? Testing predictions from the default-mode interference hypothesis. Atten Def Hyp Disord.

[72] Sonuga-Barke EJS, Castellanos FX (2007). Spontaneous attentional fluctuations in impaired states and pathological conditions: a neurobiological hypothesis. Neurosci Biobehav Rev.

[73] Feige B, Biscaldi M, Saville CWN (2013). On the temporal characteristics of performance variability in attention deficit hyperactivity disorder (ADHD). PLoS ONE.

[74] Mowinckel AM, Alnæs D, Pedersen ML (2017). Increased default-mode variability is related to reduced task-performance and is evident in adults with ADHD. Neuroimage Clin.

[75] Zhang J, Cheng W, Liu Z (2016). Neural, electrophysiological and anatomical basis of brain-network variability and its characteristic changes in mental disorders. Brain.

[76] Barber AD, Jacobson LA, Wexler JL (2014). Connectivity supporting attention in children with attention deficit hyperactivity disorder. Neuroimage Clin.

[77] Nomi JS, Schettini E, Voorhies W (2018). Resting-state brain signal variability in prefrontal cortex is associated with ADHD symptom severity in children. Front Hum Neurosci.

[78] Levy F (1991). The dopamine theory of attention deficit hyperactivity disorder (ADHD). Aust N Z J Psychiatry.

[79] Li D, Sham PC, Owen MJ, He L (2006). Meta-analysis shows significant association between dopamine system genes and attention deficit hyperactivity disorder (ADHD). Hum Mol Genet.

[80] Dougherty DD, Bonab AA, Spencer TJ (1999). Dopamine transporter density in patients with attention deficit hyperactivity disorder. Lancet.

[81] Tripp G, Wickens JR (2008). Research Review: dopamine transfer deficit: a neurobiological theory of altered reinforcement mechanisms in ADHD. J Child Psychol Psychiatry.

[82] Tomasi D, Volkow ND, Wang R (2009). Dopamine transporters in striatum correlate with deactivation in the default mode network during visuospatial attention. PLoS ONE.

[83] Cole DM, Beckmann CF, Oei NYL (2013). Differential and distributed effects of dopamine neuromodulations on resting-state network connectivity. NeuroImage.

[84] Levy F, Pipingas A, Harris EV (2018). Continuous performance task in ADHD: is reaction time variability a key measure?. Neuropsychiatr Dis Treat.

[85] Advokat C (2010). What are the cognitive effects of stimulant medications? Emphasis on adults with attention-deficit/hyperactivity disorder (ADHD). Neurosci Biobehav Rev.

[86] Goto Y, Grace AA (2005). Dopaminergic modulation of limbic and cortical drive of nucleus accumbens in goal-directed behavior. Nat Neurosci.

[87] Goto Y, Otani S, Grace AA (2007). The Yin and Yang of dopamine release: a new perspective. Neuropharmacology.

[88] Arnsten AFT, Pliszka SR (2011). Catecholamine influences on prefrontal cortical function: relevance to treatment of attention deficit/hyperactivity disorder and related disorders. Pharmacol Biochem Behav.

[89] Gamo NJ, Wang M, Arnsten AFT (2010). Methylphenidate and atomoxetine enhance prefrontal function through α2-adrenergic and dopamine D1 receptors. J Am Acad Child Adolesc Psychiatry.

[90] Bushe CJ, Savill NC (2014). Systematic review of atomoxetine data in childhood and adolescent attention-deficit hyperactivity disorder 2009-2011: focus on clinical efficacy and safety. J Psychopharmacol (Oxford).

[91] Clemow DB, Bushe C, Mancini M (2017). A review of the efficacy of atomoxetine in the treatment of attention-deficit hyperactivity disorder in children and adult patients with common comorbidities. Neuropsychiatr Dis Treat.

