# Supplementary material for: A novel approach to intra-individual performance variability in ADHD
Source: Eur Child Adolesc Psychiatry. 2020 May 14;30(5):733–45. doi: 10.1007/s00787-020-01555-y (PMC8060200; doi:10.1007/s00787-020-01555-y)
Supplement: Supplementary file 1 — Supplementary material 1 (DOCX 12 kb) [file 787_2020_1555_MOESM1_ESM.docx]

**A novel approach to intraindividual variability in ADHD**

**Supplementary Materials**

**Magnitude of time window over/underestimation**

Separate analyses were conducted to examine to what extent participants’ reaction times diverted from the target of 1200ms. The analysis of magnitude of overestimation (F(2,185) = 20.227, p<0.001, ηp2=0.18) as well as the magnitude of RT underestimation showed a significant main effect of Group (F(2,185) = 16.296, p<0.001, ηp2=0.15). Further Bonferroni-corrected post hoc tests showed that patients with ADD (277 ± 142 ms; p<0.001) and those with ADHD (263 ± 121 ms; p<0.001) had significantly higher magnitude of RT underestimation as compared to healthy controls (156 ± 55 ms). Yet, no differences in RTSD were found between ADD and ADHD patients (t(141)=-.627, p=.531). The same pattern was found for overestimation with higher magnitude of RT overestimation for patients with ADD (304 ± 159 ms; p<0.001) and those with ADHD (292 ± 129 ms; p<0.001) as compared to healthy controls (156 ± 62 ms). This pattern basically reflects what has been reported for accuracy and for RT (which we have now included in the results section). Especially it shows that, on average, the healthy controls were on average successfully able to respond within 200ms above/below the target time. This was not the case in either of the patient groups. However, these results need to be interpreted with caution since they are only based only average values and do not give sufficient information about the reaction time pattern.
